# Peripartum Cardiomyopathy Associated With Gestational Transient Thyrotoxicosis and Severe Preeclampsia: A Case Report

**DOI:** 10.7759/cureus.93921

**Published:** 2025-10-06

**Authors:** Mélissa Denis, Violaine L Mincke, Elsa Huart, Julie Nagel, Kris G Poppe

**Affiliations:** 1 Department of Obstetrics and Gynecology, Centre Hospitalier Universitaire Saint-Pierre University Hospital, Université Libre de Bruxelles, Brussels, BEL; 2 Department of Obstetrics and Gynecology, Erasme Hospital, Université Libre de Bruxelles, Brussels, BEL; 3 Department of Endocrinology, Centre Hospitalier Universitaire Saint-Pierre University Hospital, Université Libre de Bruxelles, Brussels, BEL

**Keywords:** chorionic gonadotropin, hyperemesis gravidarum, preeclampsia, pregnancy-associated cardiomyopathy, pregnancy-associated thyrotoxicosis

## Abstract

Human chorionic gonadotropin (hCG) exerts a thyrotropic effect and contributes to the pathophysiology of gestational transient thyrotoxicosis (GTT), a typically benign, self-limiting form of hyperthyroidism that usually resolves by the late first or early second trimester of pregnancy, which is generally considered to have minimal obstetric or fetal consequences. However, identifying hyperthyroidism in pregnancy remains essential, as maternal and fetal complications may occur depending on the etiology and the presence of overt hyperthyroidism. Thyroid hormones significantly influence cardiovascular function. Although the pathophysiology of preeclampsia (PE) is not fully understood, it is associated with cardiovascular dysfunction, and several studies have explored the relationship between thyroid dysfunction and PE with inconsistent findings.

In this context, we report the case of a 30-year-old Angolan woman in the first trimester who was admitted for hyperemesis gravidarum. Laboratory tests revealed a markedly elevated hCG level (271,215 IU/L) for her gestational age. After exclusion of other causes, GTT was diagnosed, with normalization of hCG levels by 18 weeks of gestation. At 35 weeks, she developed gestational hypertension, managed with close monitoring. At 38 weeks, she presented with acute respiratory failure and hemodynamic pulmonary edema in the setting of PE and peripartum cardiomyopathy.

This case highlights that GTT, though usually benign, can lead to severe maternal complications when associated with extreme hCG levels. It also illustrates the coexistence of PE and peripartum cardiomyopathy, a rare and diagnostically challenging situation, in which distinguishing hypertensive heart failure of pregnancy from primary cardiomyopathy proved particularly difficult. These findings underscore the importance of early recognition, close monitoring, and further research to clarify the cardiovascular impact of hCG-mediated thyroid dysfunction in pregnancy.

## Introduction

Gestational transient thyrotoxicosis (GTT) is a benign, self-limiting form of hyperthyroidism occurring in early pregnancy, caused by the thyrotropic effect of elevated human chorionic gonadotropin (hCG) [1,2]. We report a case of GTT with markedly elevated hCG persisting into the early second trimester, later complicated by gestational hypertension, progression to preeclampsia (PE), and concomitant peripartum cardiomyopathy (PPCM). This unusual coexistence raises the question of whether thyroid dysfunction and elevated hCG contributed to the cardiovascular complications, an issue of clinical relevance for early recognition and prevention of severe outcomes.

## Case presentation

A 30-year-old Angolan woman, gravida three para two, presented at eight weeks’ gestation with severe vomiting, palpitations, tremors, sweating, and fatigue. She had no relevant medical history and two previous uncomplicated deliveries. On examination, she was tachycardic (100-120 beats/minute), normotensive (blood pressure (BP) of 118/69 mmHg), and afebrile.

Laboratory tests showed severe hyperthyroidism (suppressed thyroid-stimulating hormone (TSH), elevated FT4/FT3), electrolyte disturbances, acute renal insufficiency, and markedly elevated hCG. Thyroid function and hCG values are presented in Table 1.

**Table 1:** Initial laboratory values at admission compared to reference ranges for non-pregnant and first-trimester pregnant women. Markedly suppressed TSH with elevated free T4 and T3 values indicated. The serum hCG level was significantly elevated for gestational age. *: Reference values from Veltri et al. [3]. **: Gestational-age-specific hCG reference ranges established in our laboratory using the Elecsys® HCG+β assay. Roche Diagnostics, Method Sheet No. 03271749190, Version 7.0, 2023. TSH = thyroid-stimulating hormone; hCG = human chorionic gonadotropin

Parameter	Patient’s value	Normal values (non-pregnant)*	Normal values (pregnancy - first trimester)
TSH (mU/L)	<0.01	0.27–4.2	0.05–3.5
Free T4 (pmol/L)	>100	11.9–21.5	12–18.02*
Free T3 (pmol/L)	31.10	3.1–6.8	3.1–6.8
hCG (IU/L)	271,215	<5	32,065–149,571**

**Figure 1:**
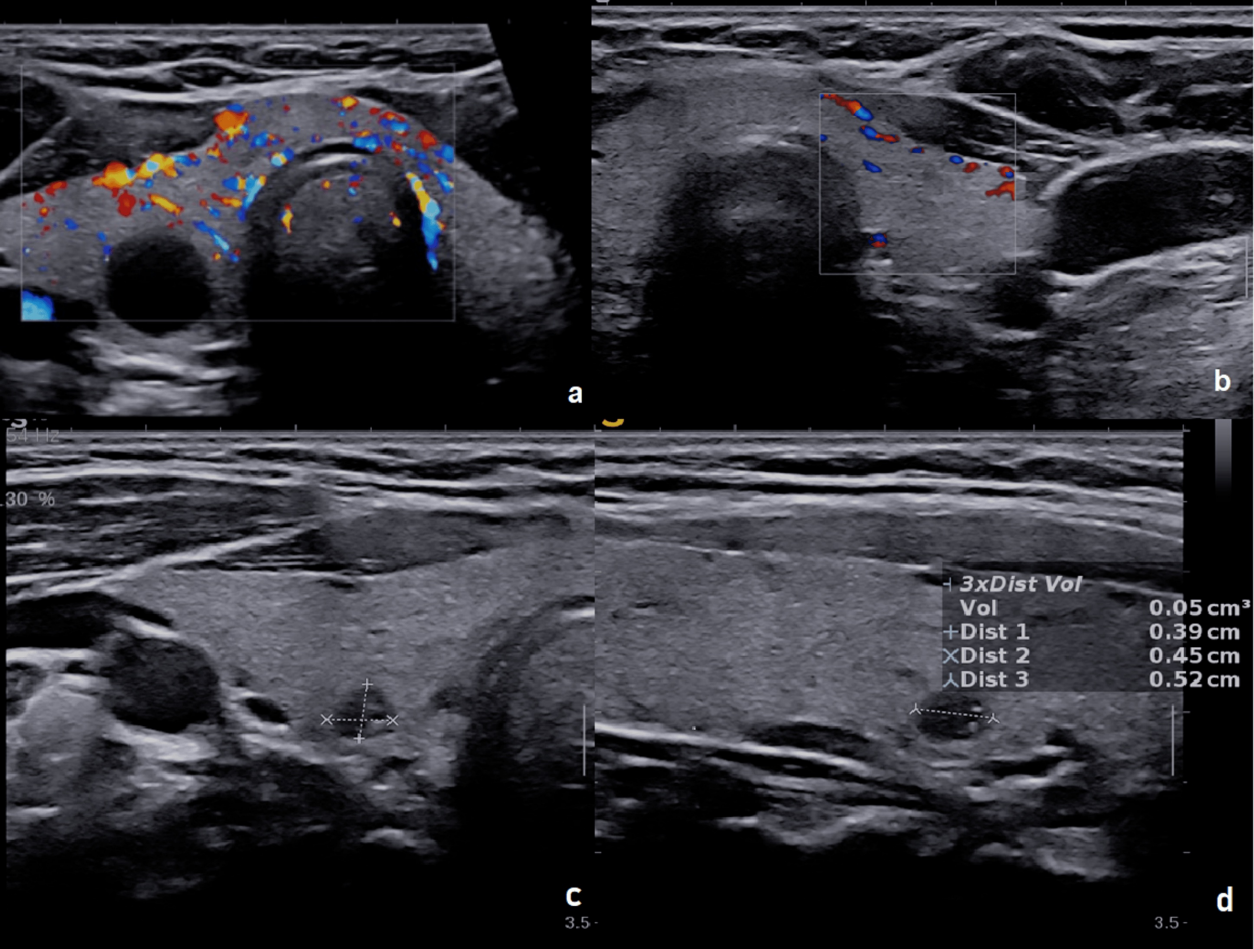
Thyroid ultrasounds. (a) Axial thyroid ultrasound showing diffuse vascular hyperemia, consistent with acute thyroiditis, and a benign cystic lesion with fine non-vascularized septations (14 × 13 × 11 mm) in the inferior pole of the right thyroid lobe (EU-TIRADS 3). (b) Axial thyroid ultrasound with color Doppler performed six weeks later showing normal vascularization. (c, d). Axial ultrasound with color Doppler performed six weeks later showing regression of the cyst (3.9 × 4.5 × 2.5 mm) and reclassification as EU-TIRADS 2.

Hyperemesis gravidarum was diagnosed, and standard treatment was initiated (intravenous (IV) fluids, antiemetics, and electrolyte replacement). Further evaluation for hyperthyroidism revealed no family or personal history of thyroid disorders. The thyroid was slightly enlarged, non-tender, with no ophthalmopathy. Thyroid function tests were negative for anti-thyroid peroxidase, anti-TSH receptor antibody (TRAb), and anti-thyroglobulin antibodies; thyroglobulin levels were normal. Thyroid ultrasound showed a small goiter with features of acute thyroiditis and a right inferior pole cyst with fine septations, which was classified as benign given its spontaneous regression within six weeks (Figure 1). These findings were considered incidental and not related to the diagnosis of GTT.

**Figure 2:**
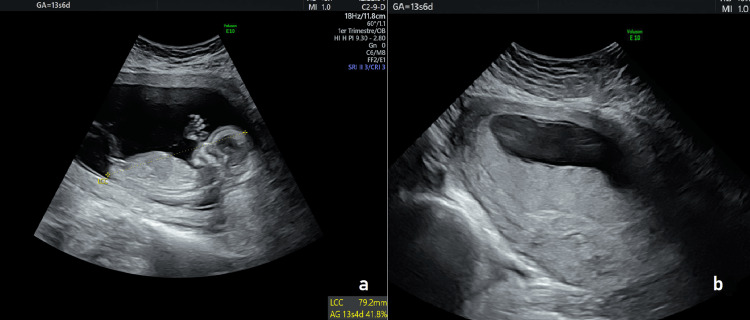
First-trimester obstetric ultrasounds. (a) Longitudinal crown–rump view showing a single intrauterine gestation. (b) Placenta with normal shape and location, without evidence of abnormalities.

Obstetric imaging excluded multiple gestation, ectopic pregnancy, and ovarian or placental abnormalities (Figure 2).

**Figure 3:**
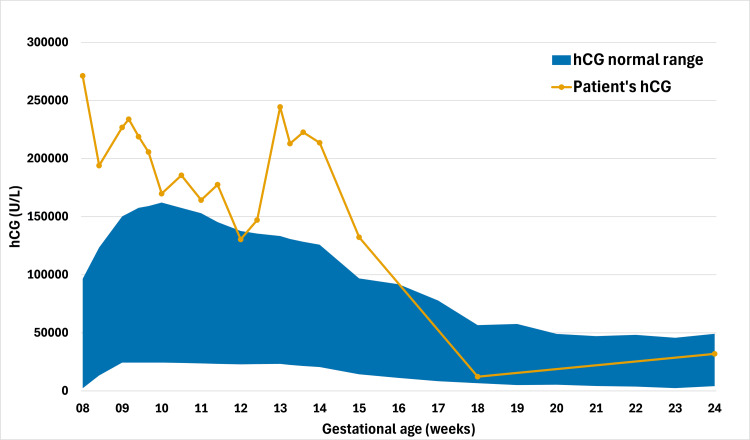
Serum hCG levels during pregnancy. Serum hCG concentrations from the first to the second trimester. The orange line represents the patient’s hCG trajectory, with abnormally high levels at eight weeks of gestation, followed by progressive normalization by 18 weeks. The blue area indicates the normal reference range (P2.5–P97.5) derived from the Generation R Study [4]. hCG = human chorionic gonadotropin

GTT was diagnosed; Graves’ disease (GD) was unlikely. Propranolol (10 mg three times daily) was started for palpitations, but as symptoms worsened (100-125 beats/minute), propylthiouracil (PTU, 100 mg twice daily) was initiated at 10 weeks due to the severity of the clinical presentation and concern for potential progression toward thyroid storm. At 15 weeks, persistently elevated hCG (Figure 3) prompted investigation for gestational trophoblastic disease, though ultrasound showed a structurally normal placenta.

**Figure 4:**
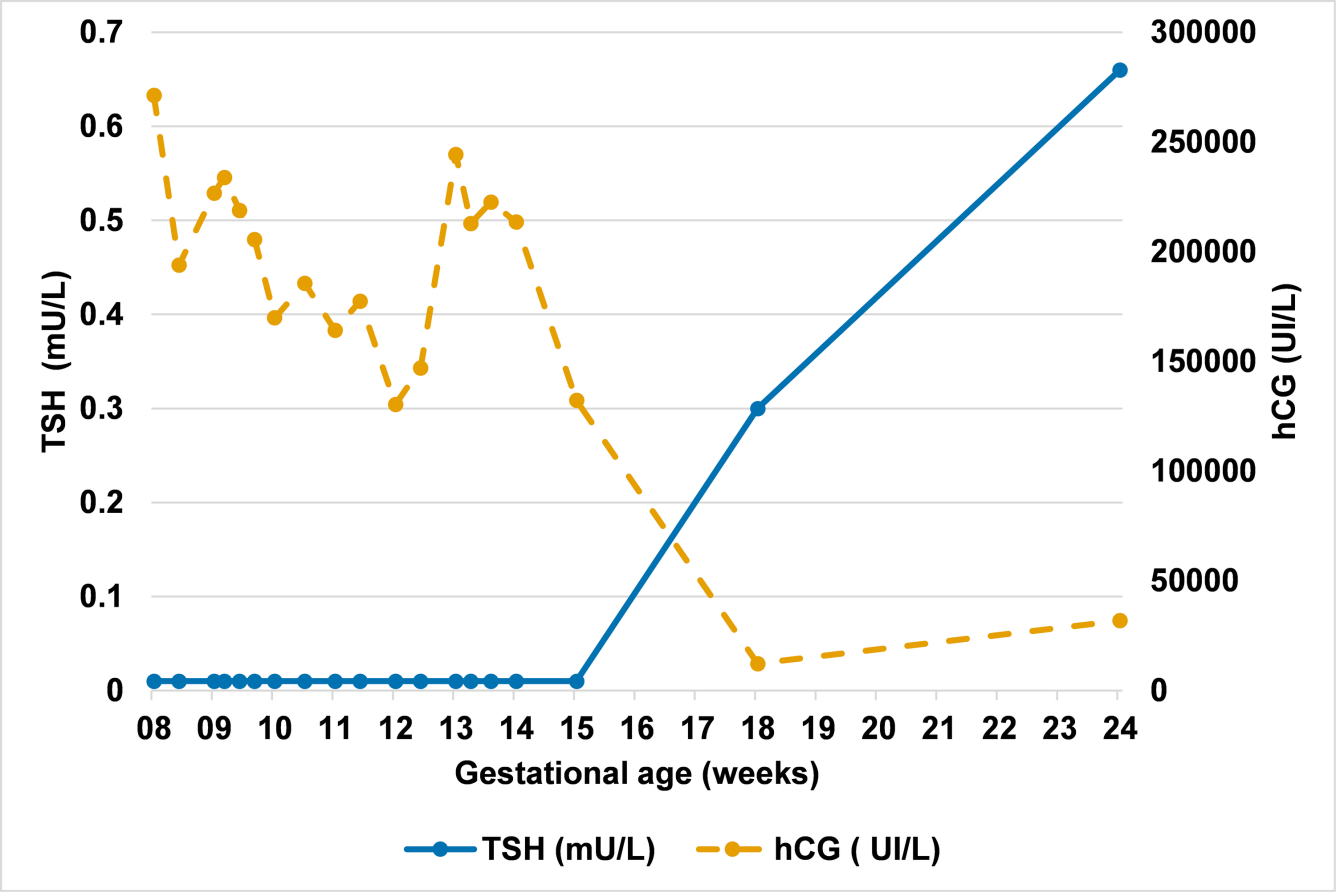
Evolution of serum TSH and hCG levels during pregnancy. TSH levels were initially suppressed in the first trimester due to the thyrotropic effect of elevated hCG, followed by normalization after 18 weeks of gestation. Antithyroid therapy was introduced at 10 weeks and discontinued at 16 weeks. The points are connected by straight line segments without interpolation (no smoothing applied). The apparent fluctuation in hCG may reflect biological and/or analytical variability. No strong physiopathological conclusion should be drawn given the limited number of data points. TSH = thyroid-stimulating hormone; hCG = human chorionic gonadotropin

As metastases commonly involve the vagina and lungs, pelvic examination and chest X-ray were performed, with negative findings. PTU was switched to methimazole (5 mg twice daily) due to hepatotoxicity risk. By 16 weeks, thyroid function normalized, allowing discontinuation of all treatments; hCG normalized by 18 weeks (Figure 4).

**Figure 5:**
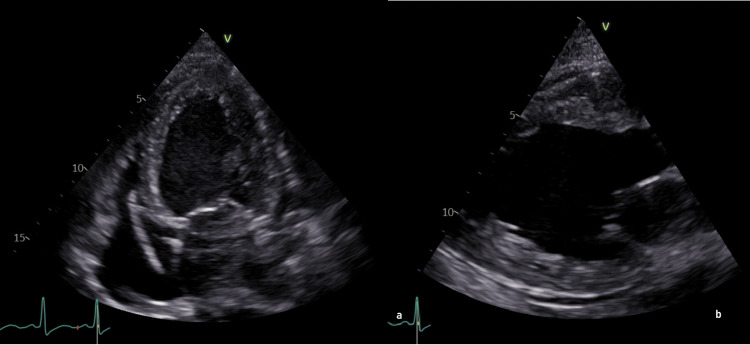
Transthoracic echocardiography. (a) Apical four-chamber view showing dilated cardiomyopathy. (b) Parasternal long-axis view illustrating dilated left ventricle.

At 35 weeks, gestational hypertension was diagnosed (BP 140/90 mmHg, no proteinuria). The patient was monitored weekly with BP, urine dipstick, and fetal surveillance. As her values never exceeded 140/90 mmHg, with diastolic pressure remaining around 85 mmHg, no antihypertensive treatment was initiated, consistent with International Society for the Study of Hypertension in Pregnancy recommendations [5]. She remained asymptomatic until 38 weeks, when she presented with acute respiratory failure, malignant hypertension (220/140 mmHg), tachycardia (130 beats/minute), and severe hypoxemia (SpO₂ 70% despite 100% FiO₂). Pulmonary auscultation revealed crackles suggestive of pulmonary edema. She received IV furosemide, magnesium sulfate, labetalol, and non-invasive ventilation, but rapid hemodynamic deterioration led to intubation. An emergency cesarean section was performed for maternal instability, delivering a 2,750 g female (Apgar scores of 4, 8, and 10) who required brief continuous positive airway pressure support and recovered without complications. The mother experienced intraoperative asystole requiring five minutes of cardiopulmonary resuscitation, attributed to multifactorial causes: severe pulmonary edema with hypoxemia (PaO₂ of 77 mmHg under 100% FiO₂), hemodynamic changes after fetal extraction, and acid-base and electrolyte disorders (pH <6.75; K˖ 3.2 mmol/L). No arrhythmia was documented on intraoperative ECG monitoring, and no anesthetic complications were identified. Echocardiography demonstrated severe systolic dysfunction with a left ventricular ejection fraction (LVEF) of 30% (normal >50%) (Figure 5).

hCG and thyroid function tests were within the normal range for gestational age. N-terminal pro-B-type natriuretic peptide was markedly elevated at 4,979 ng/L (normal <125 ng/L). Collectively, these findings were compatible with severe PE and concomitant PPCM. Histologic examination of the placenta showed no evidence of gestational trophoblastic disease or abruption.

Pulmonary edema resolved rapidly in the postpartum period, after administration of IV furosemide. Hypertension was initially controlled with IV labetalol and nicardipine, followed by oral therapy including bisoprolol, sacubitril/valsartan, and spironolactone. Bromocriptine was introduced as an investigational adjuvant therapy for PPCM [6].

At one year postpartum, echocardiography showed LVEF recovery to 50%. Postpartum TRAb testing was negative, excluding GD, and thyroid hormone levels were within normal limits. The retained diagnosis was GTT complicated by severe PE and PPCM.

## Discussion

Thyroid function and hyperthyroidism in pregnancy

hCG, secreted by syncytiotrophoblasts in early pregnancy, shares structural homology with TSH and stimulates the TSH receptor, increasing thyroid hormone (T4 and T3) production and suppressing TSH, with changes paralleling hCG fluctuations [1]. While usually physiological, these changes can precipitate hyperthyroidism in susceptible women. In pregnancy, the main causes are GTT and GD [2].

GD, an autoimmune disorder, is diagnosed by TRAb testing and requires careful management because TRAb crosses the placenta and may cause fetal hyperthyroidism. By contrast, GTT is an hCG-mediated, typically benign, self-limiting hyperthyroidism that resolves by the late first or early second trimester. GTT is generally benign, although low birth weight has been reported in cases associated with hyperemesis gravidarum [2]. As palpitations are often the sole complaint, management is mainly symptomatic with beta-blockers; antithyroid drugs are avoided in the first trimester and reserved for persistent or atypical cases [2].

In this patient, negative TRAb and normalization of hCG and TSH by 18 weeks confirmed GTT. Differentiating clinical from subclinical hyperthyroidism is essential, as complications, including thyroid storm, cardiomyopathy, hypertensive disorders, pregnancy loss, prematurity, low birth weight, stillbirth, and possible neurodevelopmental impairment, have been reported in association with uncontrolled disease [7,8]. In this case, short-term antithyroid therapy was used as a precaution until hCG levels declined spontaneously.

Thyroid function and cardiovascular effects

Thyroid hormones influence cardiovascular function by increasing heart rate, myocardial contractility, and cardiac output, while promoting peripheral vasodilation and activating the renin-angiotensin-aldosterone system, which contributes to elevated arterial pressure [9]. Prolonged thyroid hormone excess can lead to cardiac hypertrophy, atrial fibrillation, or heart failure, collectively referred to as thyrotoxic cardiovascular disease [9].

During pregnancy, thyroid hormones are essential for placental development, endothelial function, and fetal growth. Although evidence is inconsistent, high free T4 or overt hyperthyroidism in early gestation has been associated with increased risk of hypertensive disorders [10]. With rising cardiovascular demand in pregnancy, uncontrolled thyrotoxicosis predisposes to heart failure, reported in up to 10% of cases [11].

Heart failure in late pregnancy

In this case, heart failure occurred at a distance from the episode of GTT, indicating that another mechanism had to be considered. A differential diagnosis was required between hypertensive heart failure of pregnancy (HHFP) and PPCM, as both arise in late pregnancy or the postpartum period. PPCM is defined as a cardiomyopathy characterized by left ventricular systolic dysfunction (LVEF <45%) without another identifiable cause of heart failure [6]. In contrast, HHFP refers to heart failure secondary to pregnancy-associated hypertension, including PE, usually presenting with preserved or moderately reduced LVEF, often in the context of diastolic dysfunction [12,13]. It occurs more often in women with personal or familial hypertension. HHFP often improves rapidly once blood pressure is controlled, whereas PPCM more frequently shows delayed or incomplete recovery, with full normalization in only about half of cases [13].

The literature remains divided. Some exclude hypertensive cases from PPCM [13], while others highlight the strong association with PE, reported in 20-37% of cases, suggesting overlapping mechanisms involving placental anti-angiogenic factors that suppress vascular endothelial growth factor signaling and contribute to vascular disease [12,14].

Several aspects of this case favored a diagnosis of PPCM over HHFP: the absence of diastolic dysfunction, the profoundly reduced LVEF, and the clinical course, all consistent with PPCM coexisting with severe PE.

Extremely high hCG levels

In this case, exceptionally high hCG levels raised concerns for multiple pregnancy, hyperplacentosis, or gestational trophoblastic disease/neoplasia. However, ultrasound demonstrated a normal placenta, hCG levels normalized during the second trimester, and postpartum histology was unremarkable.

The markedly elevated hCG also raised questions about its potential role in the pathogenesis of pregnancy-induced hypertension, particularly PE. Beyond its role in supporting the corpus luteum, hCG promotes uterine angiogenesis and modulates immune tolerance at the maternal-fetal interface [15]. Moreover, it has been suggested that abnormal levels, whether excessively high or low, could be linked to adverse pregnancy outcomes. Peris et al. reported that extreme β-hCG levels, particularly above two to three multiples of the median, are associated with an increased risk of PE [16]. While some authors suggest that elevated β-hCG levels contribute causally by impairing trophoblastic invasion [17], others interpret them as a consequence of poor placentation. Supporting this hypothesis, one study reported that PE is more likely to be associated with elevated β-hCG levels in the second trimester, whereas no difference was observed in the first trimester [18].

It is noteworthy that four isoforms of hCG exist, namely, the classical form, hyperglycosylated hCG, β-hCG, and the sulfated form. Among these, hyperglycosylated hCG and β-hCG have been specifically associated with hypertensive disorders such as PE, though clinical applications remain uncertain [19].

## Conclusions

This case illustrates the complexity of diagnosing and managing overlapping endocrine, cardiovascular, and obstetric conditions in pregnancy. Although GTT is generally benign and self-limited, the potential for thyroid hormone excess to precipitate cardiovascular complications should not be underestimated. The unusually high hCG levels observed remain unexplained and suggest that further investigation into the role of hCG isoforms in maternal cardiovascular dysfunction is needed. Furthermore, this case highlights that severe PE and PPCM can coexist, posing a rare but significant diagnostic challenge, and underscores the importance of early recognition and careful monitoring to prevent maternal and fetal complications.
